# The KIR2DL family serves as prognostic biomarkers and correlates with immune infiltrates in acute myeloid leukaemia

**DOI:** 10.1111/jcmm.18256

**Published:** 2024-03-25

**Authors:** Wenling Liu, Mingming Zhu, Ganggang Li, Yaming Xi

**Affiliations:** ^1^ The First Clinical Medical College of Lanzhou University Lanzhou China; ^2^ Affiliated Hospital of Qinghai University Xining China; ^3^ The Fifth People's Hospital of Qinghai Province Xining China; ^4^ Department of Hematology The First Hospital of Lanzhou University Lanzhou China

**Keywords:** acute myeloid leukaemia, immune infiltrate, *KIR2DL*

## Abstract

Acute myeloid leukaemia (AML) is a prevalent haematological malignancy in which various immune and stromal cells in the bone marrow microenvironment have instrumental roles and substantially influence its progression. *KIR2DL* is a member of the immunoglobulin‐like receptor family and a natural killer (NK) cell surface‐specific receptor. However, its impact on immune infiltration regarding AML has not been addressed. We aimed to explore molecular markers associated with the immune microenvironment and prognosis of AML with a particular focus on *KIR2DL* family members. Analysis of data from The Cancer Genome Atlas and Genotype‐Tissue Expression databases revealed that *KIR2DL1, KIR2DL3* and *KIR2DL4* expression were significantly upregulated in AML and associated with decreased overall survival (OS). Moreover, univariate Cox analysis implicated *KIR2DL* genes as independent prognostic markers of OS. Functional enrichment analysis revealed that *KIR2DL* genes were associated with immune cells, the immune microenvironment and NK cell‐mediated cytotoxicity. Additionally, immune infiltration analyses revealed that *KIR2DL* upregulation was associated with stronger immune infiltration. Finally, we performed drug sensitivity profiling of *KIR2DL* genes using the Cellminer database. Collectively, our findings suggest that *KIR2DL1, KIR2DL3* and *KIR2DL4* have critical roles in AML and may represent novel biomarker genes for disease prognosis and immune infiltration.

## 1 INTRODUCTION

Acute myeloid leukaemia (AML) is a phenotypically highly heterogeneous hematologic malignancy characterised by abnormal clonal expansion and stalled differentiation of haematopoietic stem cells.^1^, ^2^ AML primarily manifests as the aberrant proliferation of primitive or naive myeloid cells in peripheral blood and bone marrow (BM).^3^ The predominant treatment strategies for AML include induction‐remission chemotherapy and intensive therapy after remission; however, the overall survival (OS) of patients has not improved substantially.^4^, ^5^ Moreover, fewer than one‐third of adult AML patients experience durable remission, highlighting the need for novel therapeutic approaches.^6^ In view of the crucial function of the BM microenvironment in the occurrence and prognosis of AML, it is necessary to investigate immune‐related markers associated with this aspect.

The BM microenvironment primarily comprises immune cells, stromal cells, fibroblasts, various signalling molecules and the extracellular matrix, which considerably affects patient diagnosis and survival, as well as therapeutic sensitivity.^7^, ^8^ Infiltrating immune cells profoundly affect tumour progression and therapeutic efficacy by exerting pro‐ and anti‐tumour effects. The survival, proliferation and drug resistance of AML cells are greatly influenced by immune and stromal cells within the immunological microenvironment, which plays an eminent function in anti‐leukaemia responses and may refine the long‐term survival of patients with AML through modulation of immune responses.^9^, ^10^ Moreover, leukaemic blasts circumvent immune surveillance by undermining the immune environment, thus facilitating disease progression.^11^, ^12^

Killer cell immunoglobulin‐like receptor (KIR) is extensively distributed on the surface of natural killer (NK) cells and partially on cytotoxic T lymphocytes.^13^, ^14^ Inhibitory KIR proteins with l cytoplasmic structural domains transduce inhibitory signals through immunoreceptor tyrosine‐based inhibitory motifs (ITIMs), whereas activating KIR proteins with S cytoplasmic structural domains deficient in ITIMs transmit activating signals.^14^, ^15^, ^16^ Accordingly, the KIR‐L series, comprising *KIR2DL1*, *KIR2DL3* and *KIR2DL4*, are inhibitory receptors that can convey suppressive signals to NK cells; however, *KIR2DL4* can also elicit activating effects, as it contains an arginine tyrosine activation motif in its transmembrane region and an ITIM in its cytoplasmic tail.^14^, ^17^ Therefore, *KIR2DL1*, *KIR2DL3* and *KIR2DL4* reportedly have significant functions in several disorders.^15^

To our knowledge, the impact of KIRs on immune infiltration regarding AML has not been addressed. Therefore, we aim to explore molecular markers associated with the immune microenvironment and prognosis of AML with a particular focus on *KIR2DL* family members. We investigate the expression of *KIR2DL* family members in patients with AML and the associated relationship with disease prognosis and immune infiltration based on analysis of data from The Cancer Genome Atlas (TCGA) and Genotype‐Tissue Expression (GTEx) databases and analysis of BM samples. We think that our research offers important insights into the molecular markers of the BM immunological milieu in AML as well as predicting patient prognosis.

## 2 MATERIALS AND METHODS

### 2.1 Databases

Transcriptome profiles and clinical data of patients with acute myeloid leukaemia were obtained from TCGA (https://www.tcga.org/; *n* = 173) and GTEx (https://gtexportal. org/home/; *n* = 70). Additionally, RNA sequencing data were downloaded in TPM format from the Xena (https://xenabrowser.net/datapages/) and GTEx databases for pan‐cancer analysis.

Expression of the *KIR2DL* family members in AML and their relationship with prognosis expression profiling data (HTSeq‐Counts) were extracted to identify differentially expressed genes (DEGs) using the R package DESeq2 (*p* < 0.05 and |log2fold‐change| = 1). Subsequently, patient samples were assigned to high and low expression groups based on the median expression of *KIR2DL1, KIR2DL3* and *KIR2DL4*. Additionally, variations in *KIR2DL1, KIR2DL3* and *KIR2DL4* expression were compared between pan‐cancer and AML samples by assessing how gene expression variations impacted AML clinical parameters and prognosis.

### 2.2 Clinical samples and Real‐Time Quantitative PCR(qRT‐PCR)

BM samples of 15 AML patients and 10 healthy controls were collected from the First Hospital of Lanzhou University (Lanzhou, China), which were approved by the Ethics Committee of the First Hospital of Lanzhou University (LDYYLL‐2023‐500).

TRIzol (TaKaRa, Japan) was used to extract total RNA. Then the qRT‐PCR reaction was conducted by CFX96 real‐time PCR System (Bio‐Rad, USA) using one‐step qRT‐PCR kit (Tiangen, China). Reaction system: 2 × SYBR Green 25 μl, 25 × Enzyme Mix 2μl, 1.25 μl of each primer for the upstream and downstream, RNA 1 μl and add ddH_2_O to the total volume of 50 μl. Reaction programme: reverse transcription for 30 min at 50°C, 3 min of pre‐denaturation at 95°C, 15 s of denaturation at 95°C, annealing for 30 s at 60°C and 40 cycles total. The sequences of the primers was detailed in Table S1. The relative expression of target genes was calculated using the comparative approach ($2^{-\Delta \Delta C_{t}}$), with *GAPDH* serving as the internal reference.

### 2.3 Gene set enrichment analysis

Functional enrichment analysis was performed using gene ontology (GO), Kyoto Encyclopedia of Genes and Genomes (KEGG) and gene set enrichment analysis (GSEA) with the R package clusterProfiler to identify significant functional differences among the *KIR2DL* members. The criteria for significant pathway enrichment included a normalised enrichment score >1, a *p*‐value <0.05 and a false discovery rate *q* < 0.05.

### 2.4 Immune infiltration analysis

The ESTIMATE algorithm was applied to assess stromal and immune cell scores in the high and low *KIR2DL1, KIR2DL3* and *KIR2DL4* expression groups. CIBERSORT is a bioinformatics method that evaluates the composition of immune cells based on the expression of standardised genes. The abundance of immune and stromal cell populations was calculated based on the known gene expression levels of immune cells by applying the single‐sample gene set enrichment analysis (ssGSEA) method using the R package GSVA and xCell algorithms.

### 2.5 Protein–protein interaction network

To identify proteins that interact with *KIR2DL* family members, we constructed a protein–protein interaction (PPI) network using the online database GeneMANIA (http://genemania.org/).

### 2.6 Drug sensitivity analysis

Drug sensitivity analysis of the *KIR2DL1, KIR2DL3* and *KIR2DL4* genes was performed using the online database CellMiner (https://discover.nci.nih.gov/cellminer/home.do). Briefly, 218 drugs approved by the FDA and 574 drugs in clinical trials were selected for analysis; *p* < 0.05 was the criterion for screening drugs.

## 3 RESULTS

### 3.1 The expression of *KIR2DL* family genes is upregulated in AML

**FIGURE 1:**
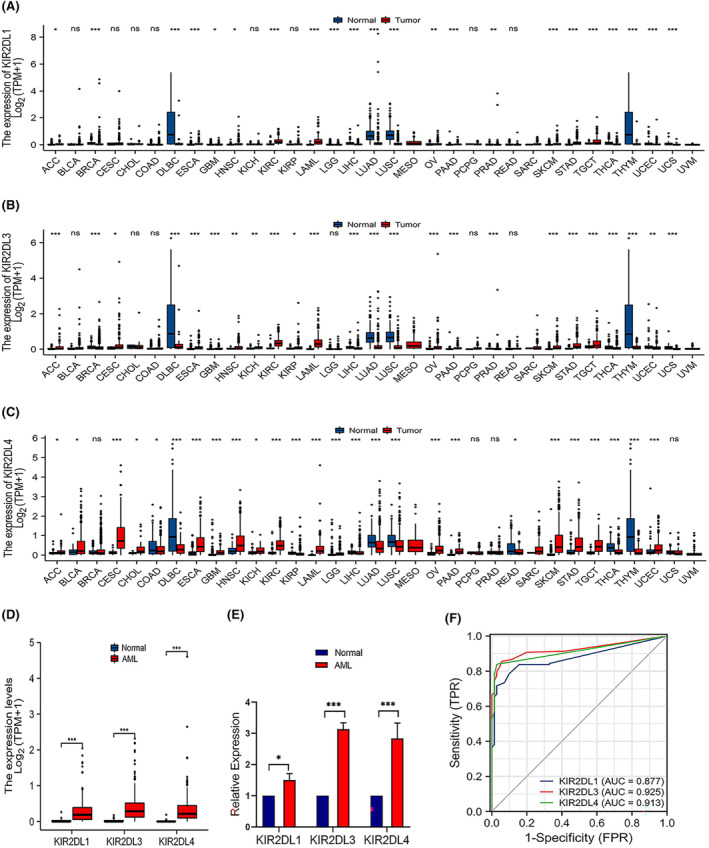
Expression levels of *KIR2DL* family members in different types of tumours and AML. (A–C) Expression of *KIR2DL1*, *KIR2DL3* and *KIR2DL4* in different types of tumours compared with normal samples in the TCGA and GTEx databases. (D) Expression of *KIR2DL1*, *KIR2DL3* and *KIR2DL4* in AML and non‐matched normal samples in the TCGA and GTEx databases. (E) Expression of *KIR2DL1*, *KIR2DL3* and *KIR2DL4* in patient with AML by qRT‐PCR. (F) ROC curves for classifying AML versus normal samples in the TCGA and GTEx databases.

The results of the pan‐carcinoma analysis suggested that the *KIR2DL* gene family was upregulated in most tumours, including adrenocortical carcinoma, bladder urothelial carcinoma, cervical squamous cell carcinoma, endocervical adenocarcinoma, oesophageal carcinoma, head and neck squamous cell carcinoma and kidney renal clear cell carcinoma. However, their expression was downregulated in breast invasive carcinoma, colon adenocarcinoma, lymphoid neoplasm, diffuse large B‐cell lymphoma and liver hepatocellular carcinoma (Figure 1A–C). Additionally, the expression levels of *KIR2DL1*, *KIR2DL3* and *KIR2DL4* were significantly higher in AML samples than those in normal samples (*p* < 0.001; Figure 1D). To clarify the expression of *KIR2DL* family genes in patient with AML, we examined their expression in clinical samples using qRT‐PCR. The results revealed that *KIR2DL1*, *KIR2DL3* and *KIR2DL4* were highly expressed in BM samples from patient with AML (Figure 1E). Furthermore, the receiver operating characteristic (ROC) curve indicated that *KIR2DL1, KIR2DL3* and *KIR2DL4* expression exhibited good predictive power to discriminate AML from normal samples, with area under the curve values of 0.877 (95% confidence interval [CI] = 0.836–0.917), 0.925 (95% CI = 0.893–0.957) and 0.913 (95% CI = 0.882–0.943), respectively (Figure 1F).

### 3.2 *KIR2DL* family expression levels correspond with AML clinical features and prognosis

The study cohort comprised 151 patients with clinical information and RNA sequencing data retrieved from TCGA. Tables S2, S3 and S4 show the clinical characteristics of patients with AML and their relationships with *KIR2DL1, KIR2DL3* and *KIR2DL4* expression levels. The *KIR2DL1* and *KIR2DL3* high expression groups comprised a larger proportion of older patients (> 60 years; *p* < 0.01) and higher white blood cell (WBC) counts (×10^9^/L), bone marrow (BM) blasts (%) and peripheral blood (PB) blasts (%) than the low expression groups (*p* < 0.05; Tables S2 and S3). Similarly, the WBC (×10^9^/L), BM blast (%) and PB blast (%) differed considerable between the *KIR2DL4* high and low expression groups (Table S4). *KIR2DL1, KIR2DL3* and *KIR2DL4* expression were associated with patient age, BM blasts (%) and WBC (×10^9^/L). More specifically, the expression of *KIR2DL1, KIR2DL3* and *KIR2DL4* increased as age increased.

**FIGURE 2:**
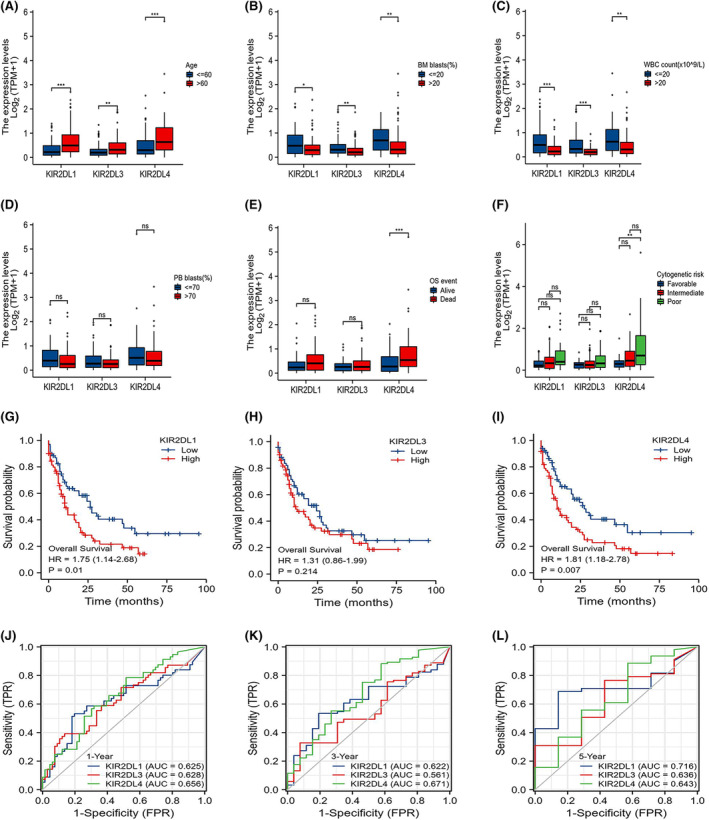
Relationship between the expression of *KIR2DL* family members and clinical features of patients with AML. (A–D) Expression of *KIR2DL1, KIR2DL3* and *KIR2DL4* is related to patient age, BM blasts (%) and WBC count (×10^9^/L). (E–F) Relationship between *KIR2DL4* and overall survival (OS) and cytogenetic risk. (G–I) Kaplan–Meier survival curves for *KIR2DL1, KIR2DL3* and *KIR2DL4* in patients with AML.

However, as BM blasts (%) and WBC (×10^9^/L) increased, the levels of *KIR2DL1, KIR2DL3* and *KIR2DL4* decreased compared with the low expression groups (*p* < 0.05; Figure 2A–D). In addition, the subgroup analysis of cytogenetic risk revealed a significant difference between the *KIR2DL4* high and low expression groups in the intermediate and poor prognosis groups. That is, high *KIR2DL4* expression was associated with intermediate and poor prognosis. *KIR2DL4* expression was also associated with the French‐American‐British (FAB) classification of AML, *FLT3* mutation and overall events.

Kaplan–Meier survival curves revealed the impact of indicators on the OS of patients. *KIR2DL4* expression was associated with OS and cytogenetic risk (Figure 2E,F). Additionally, OS was considerably lower in the *KIR2DL1* and *KIR2DL4* high expression groups (*p* = 0.01, *p* = 0.007, respectively) than in the low expression groups (Figure 2G,I). However, *KIR2DL3* expression did not impact OS (Figure 2H). Time‐dependent ROC curves were used to assess the predictive abilities of the *KIR2DL* family members. Although the assessment of the five‐year OS was slightly less favourable, the *KIR2DL* gene family exhibited significant predictive potential for the one‐ and three‐year OS (Figure 2J–L).

Univariate Cox analysis was performed (Table S5) and forest plots created to further assess whether *KIR2DL* family expression represented independent variables for OS in patients with AML (Figure S1). *KIR2DL1, KIR2DL3* and *KIR2DL4* were confirmed as separate OS predictors.

### 3.3 GO, KEGG and GSEA functional enrichment analysis

**FIGURE 3:**
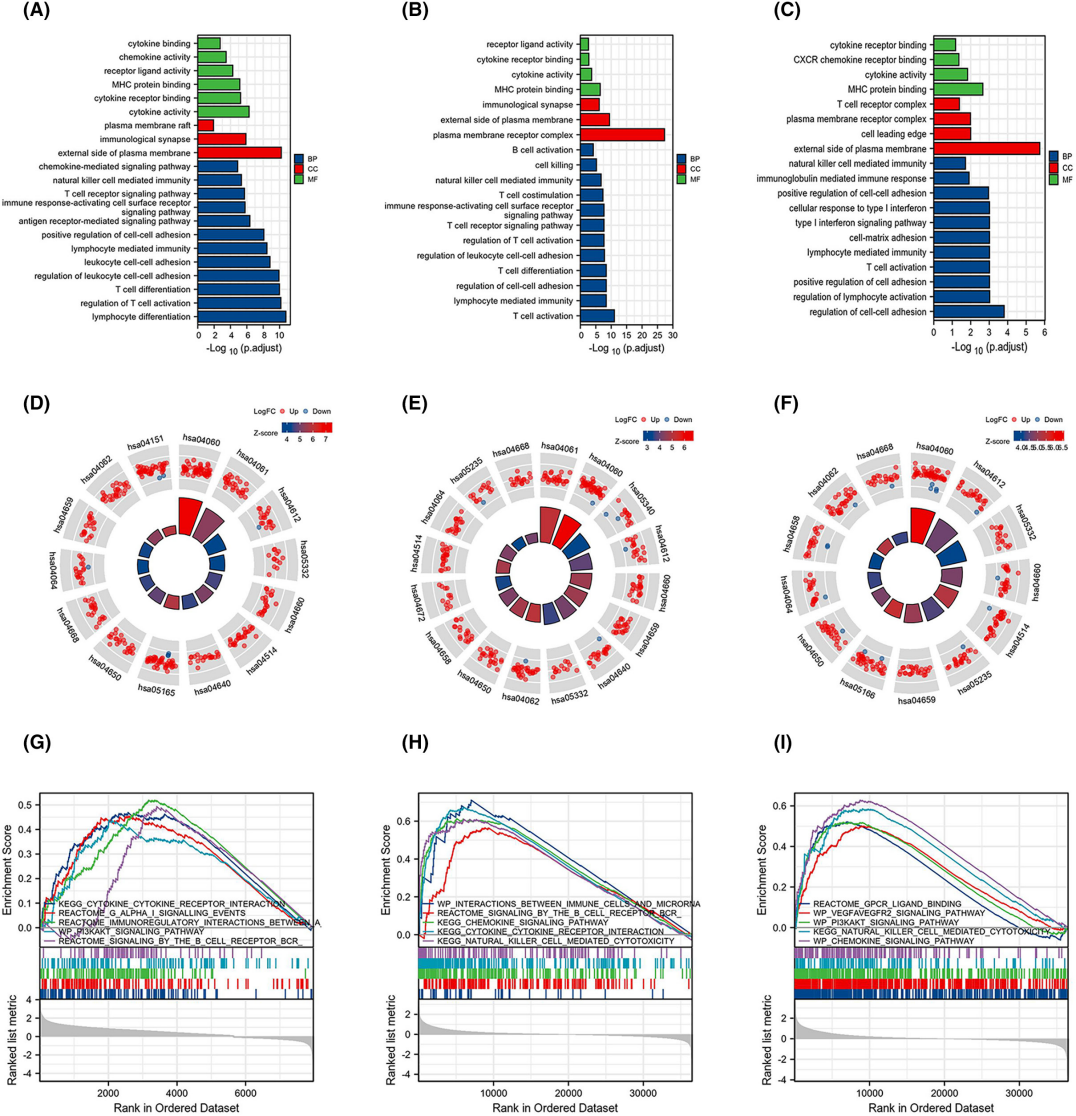
Functional enrichment analysis. (A–C) GO analysis, (D–F) KEGG pathway analysis, and (G–I) GSEA analysis of the differentially expressed genes (DEGs) based on TCGA data.

The GO enrichment analysis comprised biological processes, cellular composition and molecular functions. The DEGs of the *KIR2DL* gene family were substantially enriched in T‐cell activation and differentiation, regulation of cell–cell adhesion, lymphocyte‐mediated immunity and NK cell‐mediated immunity (Figure 3A–C). Additionally, KEGG pathway analysis showed that the significant DEG‐enriched pathways included cytokine‐cytokine receptor interactions, viral protein interactions with cytokines and cytokine receptors, T‐cell receptor signalling pathways, antigen processing and presentation and cell adhesion molecules (Figure 3D–F). Subsequently, GSEA revealed enrichment in immune cells, the immune microenvironment, cytokine receptor interactions, chemokine signalling pathways and NK cell‐mediated cytotoxicity (Figure 3G–I).

### 3.4 *KIR2DL* family expression correlates with immune infiltration

Given that functional enrichment analysis suggested that the *KIR2DL* gene family is related to immune features, we employed ESTIMATE, CIBERSORT, ssGSEA and Xcell to better characterise immune cell infiltration in the AML microenvironment.

**FIGURE 4:**
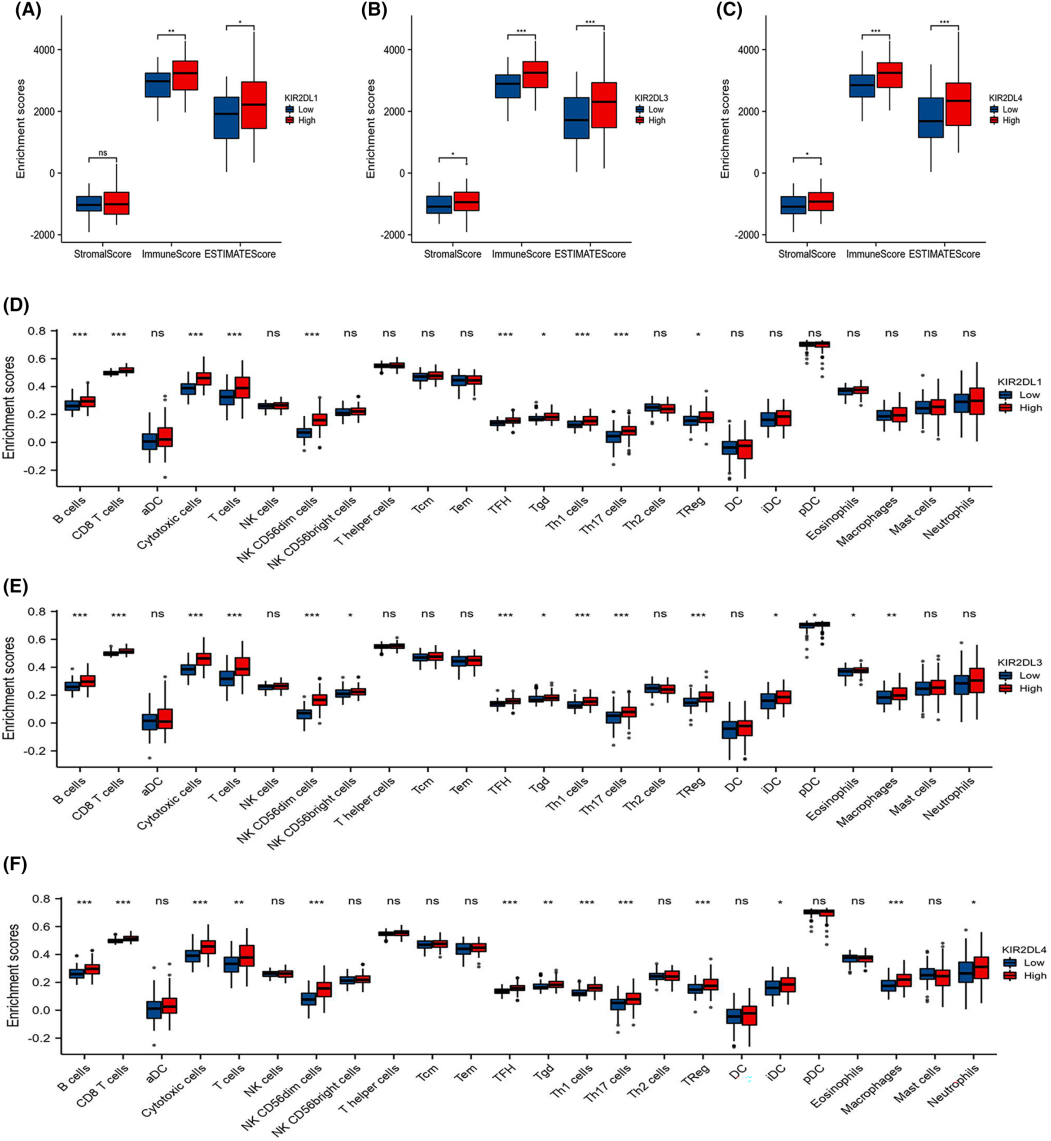
Correlation between *KIR2DL* family member expression and immune infiltration in AML. Correlation between *KIR2DL1, KIR2DL3* and *KIR2DL4* and (A–C) ESTIMATE results, (D–F) immune cell abundance as determined by ssGSEA.

The *KIR2DL1, KIR2DL3* and *KIR2DL4* high expression groups exhibited higher stromal, immune and ESTIMATE scores in the ESTIMATE analysis (Figure 4A–C). Furthermore, the ssGSEA analysis showed that the expression of *KIR2DL1*, *KIR2DL3* and *KIR2DL4* was significantly correlated with the infiltration of various immune cells, including NK CD56dim cells, cytotoxic cells, T cells, Th1 cells, CD8^+^T cells, Tfh cells, B cells, Th17 cells and Treg cells. The stronger the expression of these genes, the higher the infiltration of these immune cells (Figure 4D–F and Figure S2G–I).

Additionally, the proportion of infiltrating immune cells was assessed using the CIBERSORT algorithm (Figure S2J).

**FIGURE 5:**
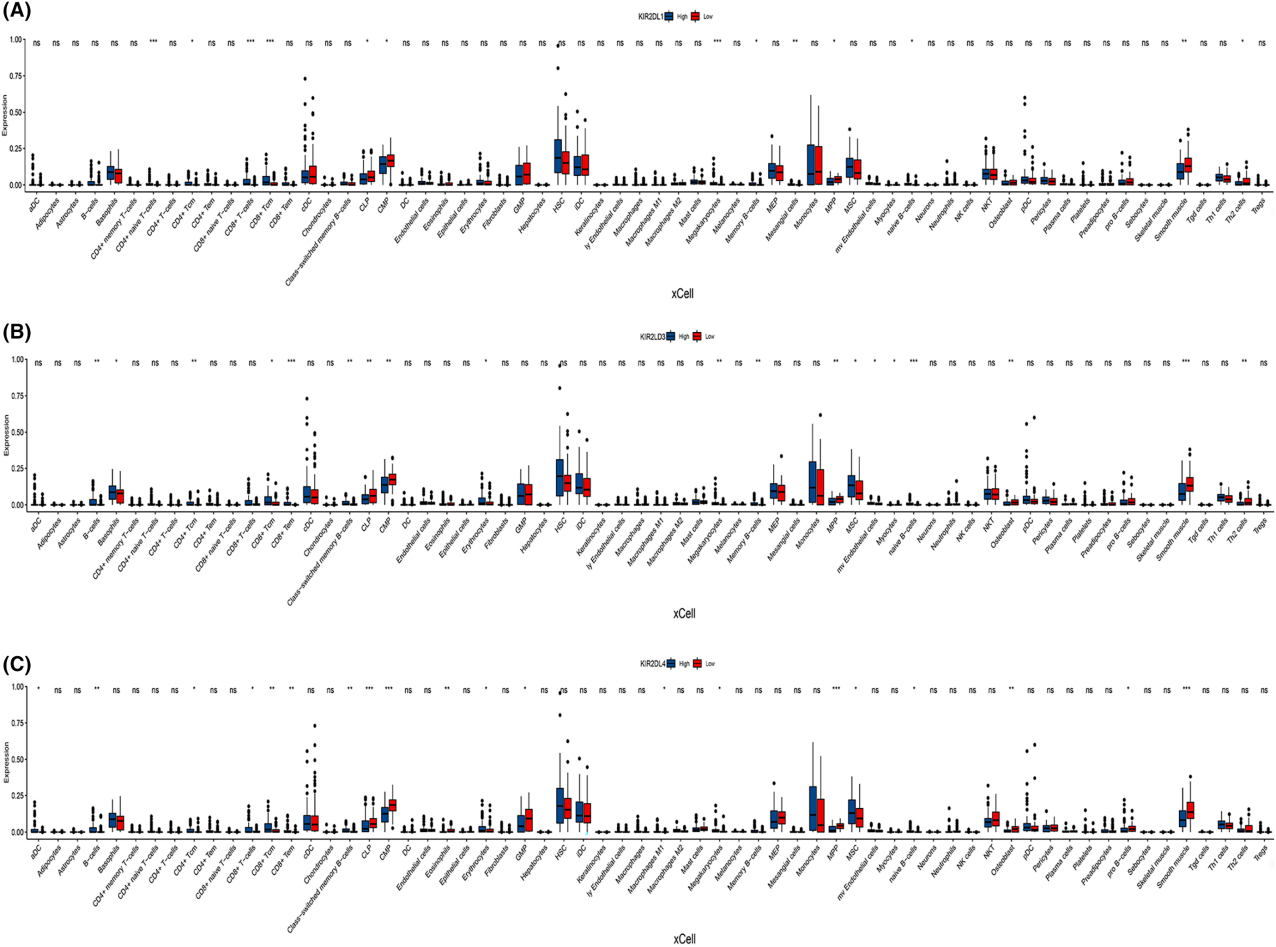
Correlation of *KIR2DL* family member expression with infiltration of 64 types of immune cells by xCell; (A) *KIR2DL1*, (B) *KIR2DL3* and (C) *KIR2DL4*.

To elucidate the relationship between the *KIR2DL* gene family and other immunocytes, we computed the levels of 64 immune cells using the xCELL algorithm. The results confirmed that the abundance of certain immune cells, including CD4^+^ T cells, CD8^+^ T cells, Th cells and B cells, was increased in the *KIR2DL* high expression group compared with those in the low expression group. Additionally, the abundance of multipotent blood progenitors (MPPs), common lymphoid progenitors (CLPs), common myeloid progenitors (CMPs), mesenchymal stem cells (MSCs), megakaryocytes, mesangial cells, osteoblasts and smooth muscle cells differed between the *KIR2DL* high and low expression groups (Figure 5A–C).

### 3.5 Protein–protein interaction (PPI) network

**FIGURE 6:**
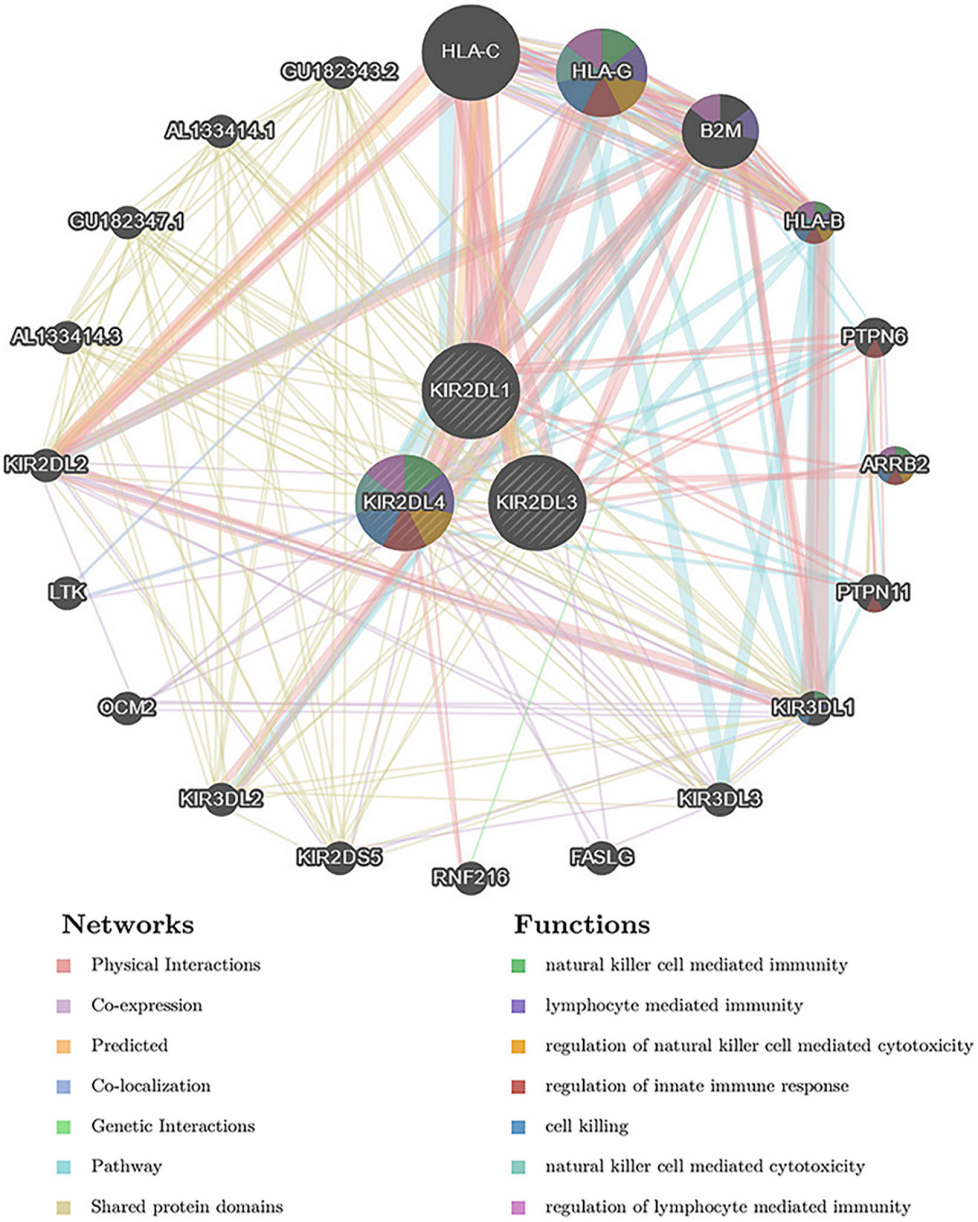
Protein–protein interaction (PPI) network of the *KIR2DL* family.

**FIGURE 7:**
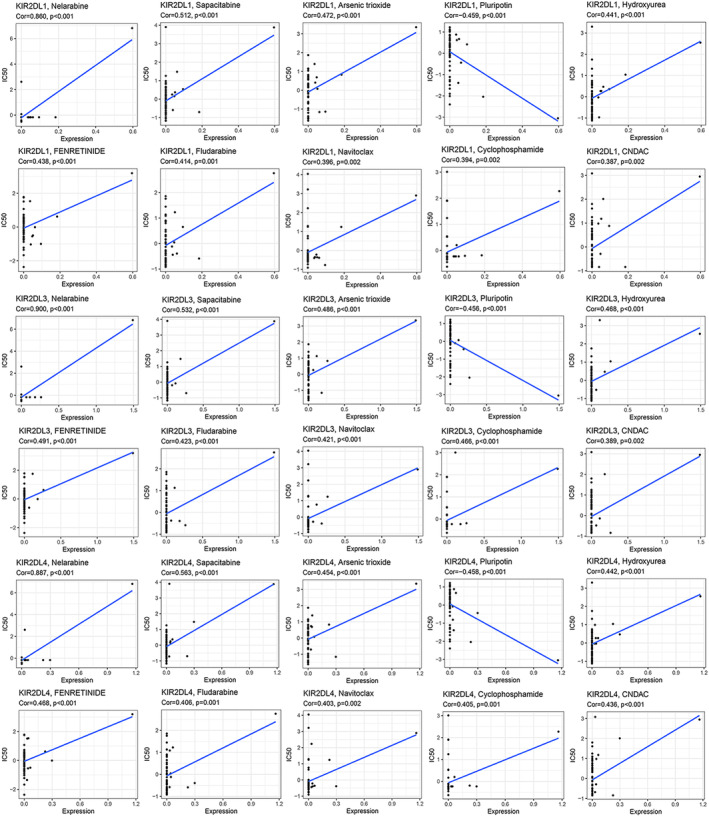
Relationship between *KIR2DL* family member expression and drug sensitivity.

The GeneMANIA database was used to explore the interactions between *KIR2DL* family members and multiple oncogenic proteins. Among the 20 proteins that interacted with *KIR2DL* proteins, HLA‐C, HLA‐G, B2M, HLA‐B, PTPN6, ARRB2 and PTPN11 were the most closely related (Figure 7). The functions of the *KIR2DL* family members and these proteins were primarily associated with NK cell‐mediated immunity, lymphocyte‐mediated immunity, regulation of innate immune response, cell killing, NK cell‐mediated cytotoxicity and regulation of lymphocyte‐mediated immunity (Figure 6).

### 3.6 *KIR2DL* family expression is associated with drug treatment sensitivity

To clarify the relationships between *KIR2DL* family expression and chemotherapeutic drug sensitivity, we assessed data within the CellMiner database related to FDA‐approved drugs as well as those tested in clinical trials. Drug sensitivity analysis revealed similar results among *KIR2DL1, KIR2DL3* and *KIR2DL4*, differing only in the COR and *p*‐values. More specifically, the sensitivity of nelarabine, sapacitabine, arsenic trioxide, hydroxyurea, fenretinide, fludarabine, navitoclax, cyclophosphamide and CNDAC was positively correlated with *KIR2DL1, KIR2DL3* and *KIR2DL4* expression. In contrast, pluripotin sensitivity was negatively correlated with *KIR2DL1, KIR2DL3* and KIR2DL4 expression (Figure 7).

## 4 DISCUSSION

In the current study, the expression of *KIR2DL1, KIR2DL3* and *KIR2DL4* was substantially upregulated in AML based on the TCGA database as well as the outcomes of qRT‐PCR. Additionally, their expression was associated with patient age, WBC count and BM blast proportion. Moreover, KIR2DL4 expression was also closely related to cytogenetic risk, FAB classification, FLT3 mutations and overall events of patients with AML. The *KIR2DL1* and *KIR2DL4* high‐expression groups also exhibited decreased OS. Univariate Cox analysis and forest plots further revealed that *KIR2DL1*, *KIR2DL3* and *KIR2DL4* represented independent predictors of OS. Collectively, these findings suggest that KIR2DL1 and KIR2DL4 significantly impact the survival of patients with AML. Inhibitory KIRs contain ITIMs, which are phosphorylated upon ligand binding, leading to the recruitment of tyrosine phosphates to inhibit NK cell killing, thereby increasing the risk of developing selected tumours.^18^ The abundance of NK cells, along with their surface inhibitory receptor *KIR2DL*, is elevated in patients with hepatoblastoma, potentially because the HLA‐C molecules on tumour cells bind to *KIR2DL* and suppress NK cytotoxicity, which leads to cancer cell immune escape.^19^ Indeed, KIR/HLA proteins are associated with susceptibility to leukaemia, colorectal cancer, cervical neoplasia and autoimmune disorders.^20^, ^21^, ^22^, ^23^ The expression of inhibitory KIR genes is correlated with leukaemia risk. That is, the high frequency of inhibitory *KIR2DL1*, *KIR2DL2* and *KIR3DL1* strongly suppresses NK cell function, thus increasing the risk of developing leukaemia.^24^ Similarly, the abundance of suppressive KIRs is reportedly increased in patients with colorectal cancer compared to healthy controls.^25^ In fact, researchers found that the absence of suppressive KIR genes prolongs the survival of patients with colorectal cancer after surgery.^26^

The results of our immune infiltration profiling indicated that the *KIR2DL1*, *KIR2DL3* and *KIR2DL4* high expression groups exhibited a comparatively high degree of infiltration in NK CD56^dim^, T and B cells compared with the low expression groups.

The NK cells are classified as CD56^dim^ CD16^+^ or CD56^bright^ CD16^−^ based on the degree of CD56 and CD16 expression and serve as the first line of defence against tumour cells.^27^ Typically, CD56^dim^ CD16^+^ NK cells account for 90% of NK cells in the peripheral blood^28^ and play a crucial role in the prevention of cancer metastasis due to their capacity to kill circulating tumour cells. In particular, NK cells help prevent AML development.^27^ A previous study also reported a higher proportion of mature NK cells with the CD56^dim^ KIR phenotype in patients with AML.^29^ A large proportion (50%) of the bone marrow NK cell population is composed of CD56^dim^ NK cells, while only a small proportion (15%) comprises CD56^bright^CD160^+^CD52^−^ NK and CD56^bright^CD160^−^CD52^+^ NK cells.^30^ When activated by target cells, CD56^dim^ CD160^+^ NK cells exhibit vigorous killing activity and interferon (IFN) secretion, whereas CD56^bright^ CD160^−^ NK cells typically play an immunoregulatory role.^27^, ^31^ Moreover, the abundances of *KIR2DL1*, *KIR2DL2* and *KIR2DL3* are higher in CD56^dim^ cells than in CD56^bright^CD160^+^CD52^−^ NK and CD56^bright^CD160^−^CD52^+^ NK cells.^30^ Romee et al.^32^ discovered that interleukin (IL)‐12, IL‐15 and IL‐18 induce memory‐like NK cells that exhibit augmented killing against AML, regardless of KIR‐ligand interactions, leading to an expanded NK cell pool of AML‐reactive effector cells. However, the microenvironment of AML not only attenuates NK cell degranulation but also upregulates inhibitory receptor expression and diminishes TNF‐α production by NK cells.^33^, ^34^

T cells are thought to reveal altered functional and phenotypic traits in the leukaemic milieu, which contribute to an immunosuppressive environment.^35^, ^36^ Traditional T helper cells that have potent immunosuppressive activity and are closely related to the progression and prognosis of tumours can differentiate into regulatory T cells (Tregs).^37^ In the current study, Tregs were more abundant in the high‐expression groups of KLR2DL family members. This agrees with the results of previous studies that reported higher proportions of Tregs in the BM of patients with AML,^38^, ^39^ and in newly diagnosed and relapsed/refractory patients with AML compared to healthy controls.^38^, ^40^, ^41^ A boosted Treg phenotype may foster disease advancement and be associated with poor prognosis in AML.^27^, ^42^, ^43^ Similarly, the frequency of Th17 cells in peripheral blood and BM mononuclear cells is substantially increased in patients with AML compared with that in healthy donors.^44^ In contrast, activated Th1 cells release IFN‐γ, a crucial mediator of innate and adaptive immunity, and may serve as an antileukaemia agent by preventing the proliferation of leukaemia cells.^45^ Patients with a high Th17 cell frequency have poor prognosis, whereas those with a high Th1 cell frequency exhibit prolonged survival.^44^ Immune defects in AML have been reflected in T‐ and NK‐cell function, with T‐cell senescence and failure, as well as impaired NK‐cell function, representing major aspects of immune dysfunction in AML patients.

The frequency of B (Breg) cells is elevated in patients with AML, which may be associated with poor prognosis.^46^ Moreover, 5% of AML cases exhibit pDC expansion (pDC‐AML), which normally has poor risk stratification.^47^, ^48^, ^49^ These results are in accordance with our findings.

Macrophages, neutrophils, eosinophils, pDC and iDC were also associated with high expression of *KIR2DL3* and *KIR2DL4*. Moreover, the *KIR2DL* high expression group exhibited high levels of CLP, CMP, megakaryocyte, mesangial cell, MPP and smooth muscle cell infiltration. In the BM microenvironment of AML, macrophage LAP restricts tumour growth and macrophages interact with leukaemic cells to promote the progression of AML by phagocytosis and immunomodulation.^50^ Consequently, high levels of *KIR2DL1, KIR2DL3* and *KIR2DL4* expression are more closely related to immune infiltrating cells, such as NK CD56^dim^ cells, T cells and B cells. Thus, the group with higher *KIR2DL* expression may be more immunologically competent.

Conventional treatment of AML has certain drawbacks, including relapse and drug resistance.^28^ However, emerging therapies, such as immunotherapy and targeted inhibitors, progressively highlight the advantages of new treatment modalities. Herein, we performed a drug sensitivity analysis for the *KIR2DL* family members using the CellMiner database. Notably, we discovered a strong relationship between the expression of *KIR2DL1, KIR2DL3* and *KIR2DL4* and chemotherapeutic agents, such as nelarabine, sapacitabine, arsenic trioxide, hydroxyurea, fenretinide, fludarabine, navitoclax and cyclophosphamide. Importantly, sensitivity was worse at higher levels of gene expression. Pluripotin may be one of the more potential agents for AML treatment. Hence, these findings present potential new targets for AML treatment.

We have verified through multiple algorithms that *KIR2DLs* may be involved in regulating the BM immune microenvironment and influencing the unfavourable prognosis of AML. There are still some limitations in the research. First, it was the result of a retrospective analysis and the number of acquired clinical specimens were relatively small. To avoid prejudice in the results, subsequently, large‐sample prospective studies should be conducted. In addition, our research results were based on public data analysis without protein‐level validation and the mechanism of *KIR2DLs* in the BM immune microenvironment. Therefore, subsequent studies on the mechanisms by which KIR2DLs restrain the immune microenvironment need to be conducted.

Collectively, the findings of the study suggest that *KIR2DL1, KIR2DL3* and *KIR2DL4* may represent independent adverse prognostic factors. The upregulated expression of *KIR2DL1, KIR2DL3* and *KIR2DL4* was closely associated with immune infiltrating cells in patients with AML. Moreover, this research offers prospective targets on the BM immune microenvironment as well as immunotherapy in AML.

## AUTHOR CONTRIBUTIONS

**Wenling liu:** Data curation (equal); methodology (equal); writing – original draft (lead). **Mingming Zhu:** Data curation (equal); methodology (equal); resources (equal). **Ganggang Li:** Data curation (equal); methodology (equal); resources (equal). **Yaming Xi:** Writing – review and editing (equal).

## FUNDING INFORMATION

This work was supported by the Natural Science Foundation of science and Technology Department of Qinghai Province (No. 2021‐ZJ‐966Q).

## CONFLICT OF INTEREST STATEMENT

The authors have no conflicts of interest to declare.

## Supporting information


Figure S1.

Figure S2.

**Tables S1–S5**.

## Data Availability

The datasets for this study are available in a public, open access repository. All data relevant to the study are included in the article or uploaded as supplementary information.
