# Supplementary figures and images for: The KIR2DL family serves as prognostic biomarkers and correlates with immune infiltrates in acute myeloid leukaemia
Source: J Cell Mol Med. 2024 Mar 25;28(8):e18256. doi: 10.1111/jcmm.18256 (PMC10963068; doi:10.1111/jcmm.18256)

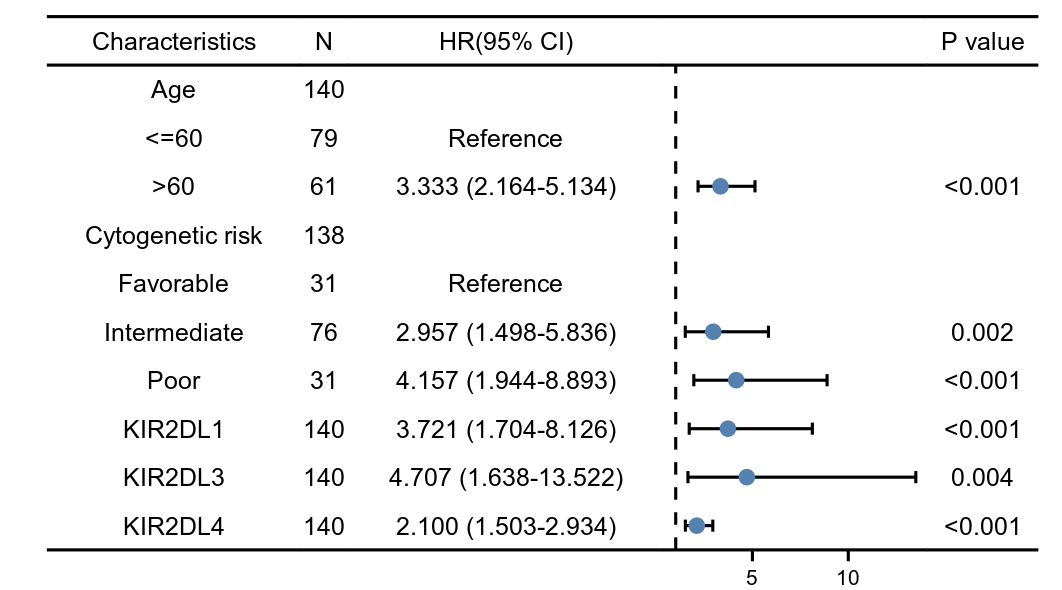

Supplement: Supplementary file 1 — Figure S1. [file JCMM-28-e18256-s003.tiff]

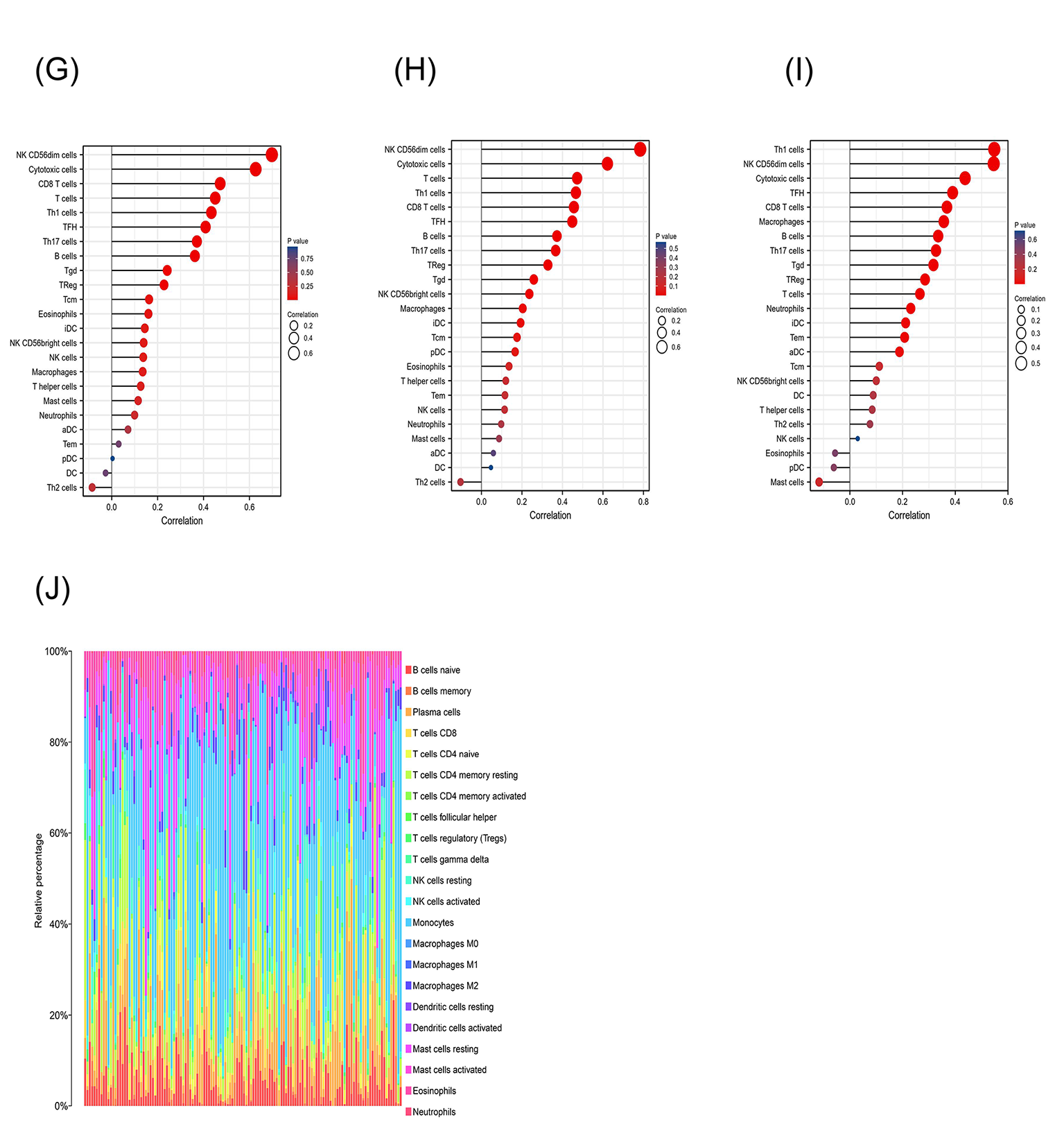

Supplement: Supplementary file 2 — Figure S2. [file JCMM-28-e18256-s002.tif]
