# Supplementary material for: The KIR2DL family serves as prognostic biomarkers and correlates with immune infiltrates in acute myeloid leukaemia
Source: J Cell Mol Med. 2024 Mar 25;28(8):e18256. doi: 10.1111/jcmm.18256 (PMC10963068; doi:10.1111/jcmm.18256)
Supplement: Supplementary file 3 — Tables S1–S5. [file JCMM-28-e18256-s001.docx]

Supplementary Material

The KIR2DL Family Serves as Prognostic Biomarkers and Correlate With Immune Infiltrates in Acute Myeloid Leukaemia

With Immune Infiltrates in Acute Myeloid Leukaemia

**Wenling Liu^1^ | Mingming Zhu^2^ | Ganggang Li^3^ | Yaming Xi^1,4^**

1 The First Clinical Medical College of Lanzhou University, Lanzhou, China

2 Affiliated Hospital of Qinghai University, Xining, China

3 The Fifth People's Hospital of Qinghai Province, Xining, China

4 Department of Hematology, The First Hospital of Lanzhou University, Lanzhou, China

**Correspondence**

Yaming Xi, Department of Hematology, The First Clinical Hospital of Lanzhou University, Lanzhou, China.

Email: [xiyaming02@163.com](mailto:xiyaming02@163.com)

## Supplementary Tables

Supplementary Table 1: Sequences of Primers for the real-time qPCR

|  | **Sequence 5’-3’** | **Product length** | **Ann. Temp.** |
| --- | --- | --- | --- |
| KIR2DL1 | F: CACGGAGGGACCTACAGATG | 141 | 60 °C |
|  | R: GGTTTTGGAGCTTGGTTCAG |  |  |
| KIR2DL3 | F: CCACTGAACCAAGCTCCGAAAC | 137 | 59 °C |
|  | R: GCACCAGCGATGAAGGAGAAAG |  |  |
| KIR2DL4 | F: GTCACTGCGTCCTGGCAGCAGAAGCTGCAC | 129 | 59 °C |
|  | R: AGGCACCAGATTTGTGGTGTGAGGAAGAGT |  |  |
| GAPDH | F: TCGGAGTCAACGGATTTGGT | 181 | 60 °C |
|  | R: TTCCCGTTCTCAGCCTTGAC |  |  |

|  |
| --- |

Supplementary Table 2. Correlation of *KIR2DL1* expression of clinical characteristics in patients with AML

|  | Low expression of KIR2DL1 | High expression of KIR2DL1 | p |
| --- | --- | --- | --- |
| n | 75 | 76 |  |
| Age, n (%) |  |  | 0.001 |
| <=60 | 54 (35.8%) | 34 (22.5%) |  |
| >60 | 21 (13.9%) | 42 (27.8%) |  |
| WBC count(x10^9/L), n (%) |  |  | 0.003 |
| <=20 | 29 (19.3%) | 48 (32%) |  |
| >20 | 46 (30.7%) | 27 (18%) |  |
| PB blasts(%), median (IQR) | 74 (56.5, 86) | 62.5 (40, 81) | 0.02 |
| BM blasts(%), median (IQR) | 49 (16, 75) | 26 (6, 54) | 0.005 |
| Cytogenetic risk, n (%) |  |  | 0.068 |
| Favorable | 20 (13.7%) | 11 (7.2%) |  |
| Intermediate | 40 (27.4%) | 42 (27.6%) |  |
| Poor | 13 (8.9%) | 23 (15.1%) |  |
| FAB classifications, n (%) |  |  | 0.639 |
| M0 | 7 (5.1%) | 8 (5.9%) |  |
| M1 | 21 (15.4%) | 14 (10.4%) |  |
| M2 | 19 (13.9%) | 19 (14.2%) |  |
| M4 | 15 (11.0%) | 14 (10.4%) |  |
| M5 | 6 (4.4%) | 9 (6.7%) |  |
| M6 | 0 (0%) | 2 (1.5%) |  |
| M7 | 0 (0%) | 1 (0.7%) |  |
| Cytogenetics, n (%) |  |  | 0.128 |
| Normal | 35 (25.7%) | 34 (25.4%) |  |
| 8 | 5 (3.7%) | 3 (2.2%) |  |
| del(5) | 1 (0.7%) | 0 (0%) |  |
| del(7) | 1 (0.7%) | 5 (3.7%) |  |
| inv(16) | 6 (4.4%) | 2 (1.5%) |  |
| t(8;21) | 5 (3.7%) | 2 (1.5%) |  |
| t(9;11) | 0 (0%) | 1 (0.7%) |  |
| Complex | 7 (5.1%) | 17 (12.7%) |  |
| FLT3 mutation, n (%) |  |  | 0.259 |
| Negative | 40 (30.3%) | 47(35.6%) |  |
| Positive | 26 (19.7%) | 19 (14.4%) |  |
| OS event, n (%) |  |  | 0.112 |
| Alive | 32 (21.3%) | 22 (14.5%) |  |
| Dead | 43 (28.7%) | 54 (35.5%) |  |

Supplementary Table 3. Correlation of *KIR2DL3* expression of clinical characteristics in patients with AML

| Characteristic | Low expression of KIR2DL3 | High expression of KIR2DL3 | p |
| --- | --- | --- | --- |
| n | 75 | 76 |  |
| Age, n (%) |  |  | 0.01 |
| <=60 | 52 (34.6%) | 36 (23.7%) |  |
| >60 | 23 (15.3%) | 40 (26.3%) |  |
| WBC count(x10^9/L), n (%) |  |  | 0.009 |
| <=20 | 30 (20%) | 47 (31.3%) |  |
| >20 | 45 (30%) | 28 (18.7%) |  |
| BM blasts(%), n (%) |  |  | 0.015 |
| <=20 | 22 (14.6%) | 38 (25%) |  |
| >20 | 53 (35.3%) | 38 (25%) |  |
| PB blasts(%), median (IQR) | 72 (58, 86) | 65.5 (40, 81.25) | 0.017 |
| Cytogenetic risk, n (%) |  |  | 0.543 |
| Favorable | 16 (10.8%) | 15 (10%) |  |
| Intermediate | 43 (29.1%) | 39 (26%) |  |
| Poor | 15 (10.1%) | 21 (14%) |  |
| FAB classifications, n (%) |  |  | 0.223 |
| M0 | 5 (3.7%) | 10 (7.5%) |  |
| M1 | 18 (13.2%) | 17 (12.7%) |  |
| M2 | 25 (18.4%) | 13 (9.7%) |  |
| M4 | 13 (9.6%) | 16 (11.9%) |  |
| M5 | 7 (5.1%) | 8 (5.9%) |  |
| M6 | 0 (0%) | 2 (1.5%) |  |
| M7 | 0 (0%) | 1 (0.7%) |  |
| Cytogenetics, n (%) |  |  | 0.258 |
| Normal | 38 (29.2%) | 31 (26.3%) |  |
| 8 | 4 (3.1%) | 4 (3.4%) |  |
| del(5) | 1 (0.8%) | 0 (0%) |  |
| del(7) | 1 (0.8%) | 5 (4.2%) |  |
| inv(16) | 5 (3.8%) | 3 (2.5%) |  |
| t(8;21) | 5 (3.8%) | 2 (1.7%) |  |
| t(9;11) | 1 (0.8%) | 0 (0%) |  |
| Complex | 10 (7.7%) | 14 (11.9%) |  |
| FLT3 mutation, n (%) |  |  | 0.308 |
| Negative | 42 (30.9%) | 45 (35.2%) |  |
| Positive | 26 (19.1%) | 19 (14.8%) |  |
| OS event, n (%) |  |  | 1 |
| Alive | 27 (18%) | 27 (17.8%) |  |
| Dead | 48 (32%) | 49 (32.2%) |  |

Supplementary Table 4. Correlation of *KIR2DL4* expression of clinical characteristics in patients with AML

| Characteristic | Low expression of KIR2DL4 | High expression of KIR2DL4 | p |
| --- | --- | --- | --- |
| n | 75 | 76 |  |
| Age, n (%) |  |  | < 0.001 |
| <=60 | 55 (36.7%) | 33 (21.7%) |  |
| >60 | 20 (13.3%) | 43 (28.3%) |  |
| WBC count(x10^9/L), n (%) |  |  | 0.001 |
| <=20 | 28 (18.7%) | 49 (32.7%) |  |
| >20 | 47 (31.3%) | 26 (17.3%) |  |
| BM blasts(%), n (%) |  |  | 0.002 |
| <=20 | 20 (13.3%) | 40 (26.3%) |  |
| >20 | 55 (36.7%) | 36 (23.7%) |  |
| PB blasts(%), median (IQR) | 73 (52.5, 86) | 70 (41.75, 83) | 0.12 |
| Cytogenetic risk, n (%) |  |  | 0.008 |
| Favorable | 22 (15.1%) | 9 (5.9%) |  |
| Intermediate | 39 (26.7%) | 43 (28.2%) |  |
| Poor | 12 (8.2%) | 24 (15.8%) |  |
| FAB classifications, n (%) |  |  | 0.002 |
| M0 | 2 (1.6%) | 13 (9.3%) |  |
| M1 | 19 (14.8%) | 16 (11.4%) |  |
| M2 | 21 (16.4%) | 17 (12.1%) |  |
| M4 | 19 (14.8%) | 10 (7.1%) |  |
| M5 | 3 (2.3%) | 12 (8.6%) |  |
| M6 | 0 (0%) | 2 (1.4%) |  |
| Cytogenetics, n (%) |  |  | 0.031 |
| Normal | 32 (23.7%) | 37 (27.6%) |  |
| 8 | 4 (3%) | 4 (3%) |  |
| del(5) | 0 (0%) | 1 (0.7%) |  |
| del(7) | 4 (3%) | 2 (1.5%) |  |
| inv(16) | 6 (4.4%) | 2 (1.5%) |  |
| t(8;21) | 5 (3.7%) | 2 (1.5%) |  |
| t(9;11) | 0 (0%) | 1 (0.7%) |  |
| Complex | 6 (4.4%) | 18 (13.4%) |  |
| FLT3 mutation, n (%) |  |  | < 0.001 |
| Negative | 31 (24.2%) | 56 (41.2%) |  |
| Positive | 33 (25.8%) | 12 (8.8%) |  |
| OS event, n (%) |  |  | 0.023 |
| Alive | 34 (22.7%) | 20 (13.2%) |  |
| Dead | 41 (27.3%) | 56 (36.8%) |  |

Supplementary Table 5. Univariate and Multivariate analysis of *KIR2DL* family members in AML patients

| Characteristics | Total(N) | Univariate analysis | |  | Multivariate analysis | |
| --- | --- | --- | --- | --- | --- | --- |
|  |  | Hazard ratio (95% CI) | P value |  | Hazard ratio (95% CI) | P value |
| Age | 140 |  |  |  |  |  |
| <=60 | 79 | Reference |  |  |  |  |
| >60 | 61 | 3.333 (2.164-5.134) | **<0.001** |  | 2.599 (1.618-4.174) | **<0.001** |
| WBC count(x10^9/L) | 139 |  |  |  |  |  |
| <=20 | 75 | Reference |  |  |  |  |
| >20 | 64 | 1.161 (0.760-1.772) | 0.490 |  |  |  |
| BM blasts(%) | 140 |  |  |  |  |  |
| <=20 | 59 | Reference |  |  |  |  |
| >20 | 81 | 1.165 (0.758-1.790) | 0.486 |  |  |  |
| PB blasts(%) | 140 |  |  |  |  |  |
| <=70 | 66 | Reference |  |  |  |  |
| >70 | 74 | 1.230 (0.806-1.878) | 0.338 |  |  |  |
| Cytogenetic risk | 138 |  |  |  |  |  |
| Favorable | 31 | Reference |  |  |  |  |
| Intermediate | 76 | 2.957 (1.498-5.836) | **0.002** |  | 2.153 (1.070-4.333) | **0.032** |
| Poor | 31 | 4.157 (1.944-8.893) | **<0.001** |  | 2.337 (1.033-5.285) | **0.042** |
| KIR2DL1 | 140 | 1.926 (1.311-2.828) | **<0.001** |  | 1.209 (0.652-2.239) | 0.547 |
| KIR2DL3 | 140 | 2.073 (1.249-3.443) | **0.005** |  | 0.878 (0.383-2.010) | 0.758 |
| KIR2DL4 | 140 | 1.676 (1.340-2.097) | **<0.001** |  | 1.327 (0.984-1.790) | 0.063 |
